# Can N Nutrition Lead to “Plant Diabetes”? The Perspective From Ammonium Nutrition and Methylglyoxal Accumulation

**DOI:** 10.3389/fpls.2022.928876

**Published:** 2022-05-31

**Authors:** Mikel Rivero-Marcos, Idoia Ariz

**Affiliations:** Department of Sciences, Institute for Multidisciplinary Research in Applied Biology (IMAB), Public University of Navarre, Campus Arrosadía, Pamplona, Spain

**Keywords:** ammonium toxicity, diabetes, glycolysis, methylglyoxal, glyoxalase system, dicarbonyl stress

## Introduction

In quantitative terms, nitrogen (N) is the most important essential mineral element for crops, and its availability is a critical factor in agricultural plant production. As a central mineral nutrient element, N influences the processes of photosynthesis, glycolysis and respiration to provide carbon (C) skeletons for the assimilation of inorganic N into amino acids, proteins, and nucleic acids, resulting in rapid cell division, growth and biomass gain (Foyer et al., 2011). The sink cells of tissues and organs are those that must receive the C skeletons or “sugars,” mainly in the form of sucrose, provided by the source leaves for metabolic maintenance. Nevertheless, the C satiety of sink organs depends on the strength of this sink, which is in turn associated with the ability to unload and metabolize the translocated sugars (Marcelis, 1996). It is in that regard that the two primary inorganic N sources for crop plants, i.e., ammonium (${\text{NH}}_{4}^{+}$) and nitrate (${\text{NO}}_{3}^{-}$), differ. While ${\text{NH}}_{4}^{+}$ is incorporated at lower energy cost into amino acids by GS/GOGAT cycle, ${\text{NO}}_{3}^{-}$ is successively reduced by nitrate- and nitrite-reductase to ${\text{NH}}_{4}^{+}$ in a highly reductant-consuming process, usually considered the second largest sink for reducing equivalents after C fixation (Noctor and Foyer, 1998; Kronzucker et al., 2001). This distinct energy requirement by these both N sources for the incorporation of ${\text{NH}}_{4}^{+}$ into N-containing organic compounds highlights the close link between N and C metabolism. Paradoxically, although the use of ${\text{NH}}_{4}^{+}$ as N source eliminates the need for N reduction in the plant cell and is an intermediate in many metabolic reactions, it often affects plant growth and development, showing an impaired phenotype which is commonly referred to as “ammonium syndrome” (Esteban et al., 2016; Liu and Von Wirén, 2017). In fact, the high accumulation of ${\text{NH}}_{4}^{+}$ in soil favored by low pH, flooding or in paddy fields, especially after ${\text{NH}}_{4}^{+}$ fertilization, is evidenced by stunted vegetative growth and decreased yield.

Consumption of sugars is necessary for the correct functioning of the cells but an excessive flux and consumption of them can cause disorders in cells of living beings, such as accelerated onset of aging (Ingram and Roth, 2011), Alzheimer's (Hipkiss, 2019) or diabetes diseases in humans (Murao et al., 2022). At this point, plant cells may also suffer severe disorders when are exposed to adverse conditions in which glycolytic flux is usually accelerated, such as those reported in hypoxia (António et al., 2016), infection of some parasites (Mazarei et al., 2003), or ${\text{NH}}_{4}^{+}$ fertilization (Borysiuk et al., 2018). It is well known that strict ${\text{NH}}_{4}^{+}$ nutrition increases the flux and concentration of mobile sugars in plants in order to guarantee provision of C skeletons for rapid ${\text{NH}}_{4}^{+}$ assimilation (Walch-Liu et al., 2000; Borysiuk et al., 2018; Jauregui et al., 2022). However, this overflow of soluble carbohydrates compounds may lead to the production and accumulation of some by-products of primary C metabolism, such as methylglyoxal (MG, CH_3_COCHO), which is in fact a toxic molecule for cells. Interestingly, MG is often found accumulated in animal cells with an accelerated glycolysis, showing the named “dicarbonyl stress”, and this same stress is showed in human cells under some disease conditions such as the type 2 diabetes (Kalapos, 2013; Allaman et al., 2015).

Here we introduce a debate about whether plants can be suffering a “diabetes-like syndrome” depending on the N nutrition management. The idea of a plant diabetes is not new and was initially proposed by Saito et al. (2011) when they identified MG as a potent photosystem I-mediated superoxides generator in spinach chloroplasts. Later and along the same lines, Takagi et al. (2014) and Shimakawa et al. (2014) discussed the possible plant diabetes by associating it with the accumulation of MG as a common metabolite of the primary pathways of sugar anabolism and catabolism. Nevertheless, given the increasing relevance of ${\text{NH}}_{4}^{+}$ nutrition for crop production in a context of elevated atmospheric CO_2_, in addition to being a less polluting alternative to the excessive use of ${\text{NO}}_{3}^{-}$ (Subbarao and Searchinger, 2021), we examine here from a new point of view the current knowledge about the glycolytic by-product MG and its link to a possible “${\text{NH}}_{4}^{+}$ diet”-mediated plant diabetes.

## Targeting Methylglyoxal-Mediated *Dicarbonyl Stress* During Ammonium Toxicity

Although well studied in medicine, MG is relatively unknown in plant science. It is a typical oxygenated short aldehyde by-product of several metabolic pathways, including glycolysis. In fact, this is the main pathway for MG formation, specifically from the products of the phosphofructokinase enzyme, i.e., glyceraldehyde 3-phosphate (GAP) and dihydroxyacetone phosphate (DHAP). The mechanism of non-enzymatic MG formation was explained by Richard (1993). Briefly, both compounds, GAP and DHAP, are highly unstable and promptly deprotonate remaining as enediolates intermediate compounds. Then, their phosphate group is rapidly eliminated, leading to formation of MG (Richard, 1993). MG is described as a reducing sugar that glycates proteins and leads to altered function or crosslinking of proteins, resulting in the so called “Advanced Glycation End-products” (AGEs; Goldin et al., 2006). These AGEs are related to several pathologies in medicine, including diabetes mellitus (type 1 and 2; Ahmed and Thornalley, 2007). Indeed, in diabetic patients, proteins of the tissues more exposed to excess circulating blood sugar are susceptible of being glycated by the enhanced glycating power of the MG in such tissues. This leads to increasing AGEs accumulation in targets tissues and organs for diabetes disease, including the kidney, retina, and atherosclerotic plaques, where major diabetes complications occur (Hammes et al., 1999). In addition, in type 2 diabetes, the declined sensitivity of cells to insulin leads to excess of it (i.e., hyperinsulinemia) and brings about a compensatory increase in rates of glycolysis in the liver, adipose tissue, and pancreatic β cells (Guo et al., 2012). The excess of sugar susceptible to suffer glycation by MG, and the enhancement of glycolysis by hyperinsulinemia, feedback the generation of MG and consequent AGEs, giving a negative loop feedback with highly detrimental effect to cells and tissues.

**Figure 1:**
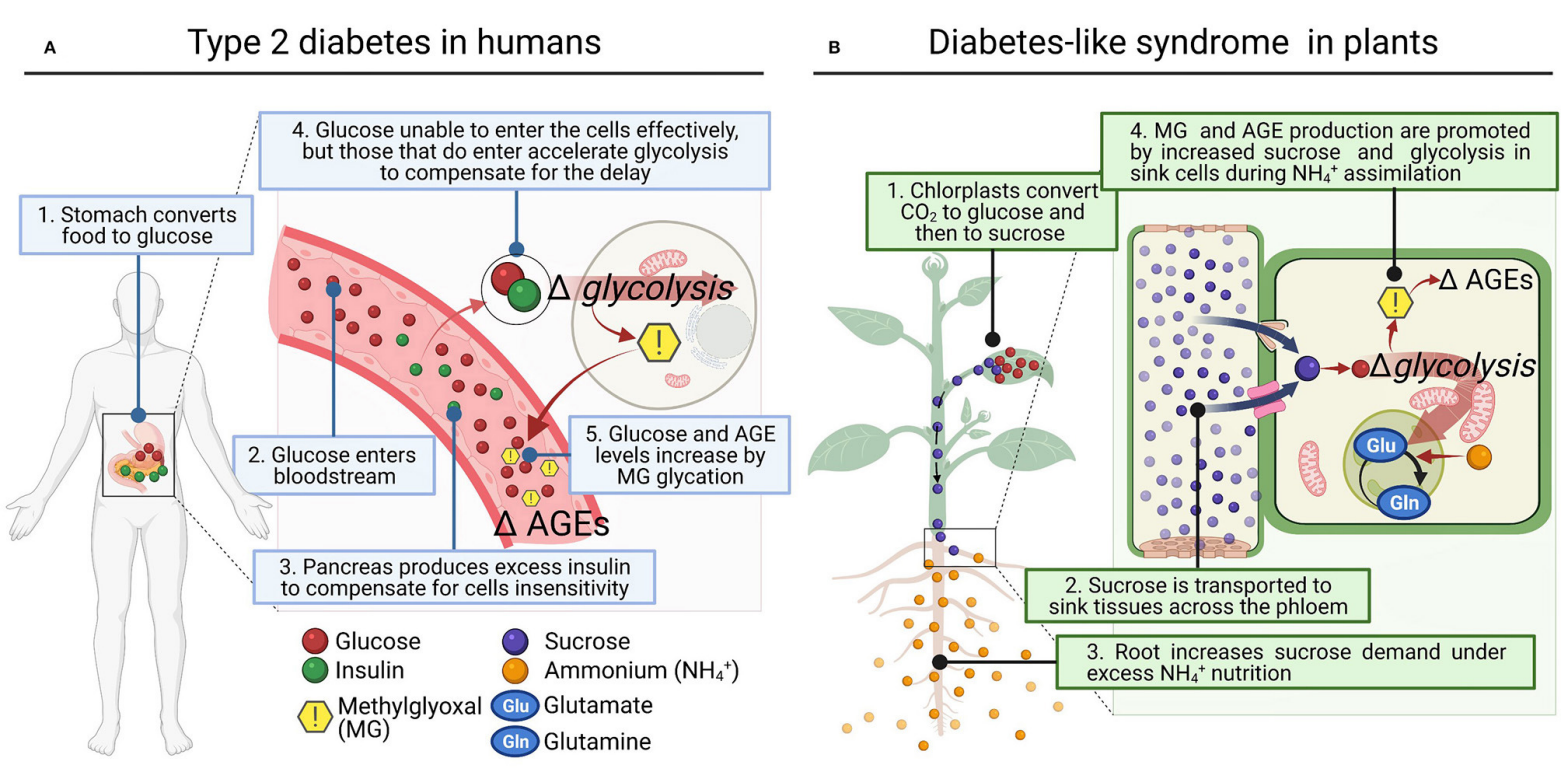
Proposed parallelism between type 2 diabetes disease in humans and diabetes-like syndrome in plants. **(A)** In the human type 2 diabetes, hyperinsulinemia results from decreased sensitivity of cells to glucose intake, and this brings in a compensatory increase in cell rates of glycolysis. **(B)** In plants subject to high external ${\text{NH}}_{4}^{+}$, roots increase the demand for sucrose, enhancing its synthesis and allocation. Consequently, the rate of glycolysis to provide C to assimilate ${\text{NH}}_{4}^{+}$ as Gln in the GS/GOGAT cycle is promoted. In both cases **(A,B)**, increased glycolysis appears to be a common response to high sugar demand by cells and MG accumulation, which induces cellular dysfunction by mediating AGEs (Advanced Glycation End-products).

At this stage, we wonder if a similar disorder could occur in plants under certain conditions that increase sugar synthesis and glycolytic flux (Figure 1). Photosynthetic organisms assimilate CO_2_ using light energy and accumulate high amounts of sugars in their cells, keeping in balance GAP and DHAP contents by equilibrium reactions during glycolysis, where MG is produced (Allaman et al., 2015). Thus, it is not surprising that higher plants, which produced vast amounts of sugars, showed significantly higher concentration of MG (i.e., 35–75 μM) than heterotrophs (2–4 μM) (Yadav et al., 2005; Rabbani and Thornalley, 2012). Interestingly, studies using spinach chloroplasts and illuminated wheat leaves under elevated CO_2_ conditions showed that MG was directly produced *via* 3-phosphoglycerate-dependent photosynthesis in a light- and time-dependent manner (Takagi et al., 2014). These studies linked an accelerated metabolic turnover of the Calvin cycle and glycolysis by enhanced photosynthesis with greater production of MG (Takagi et al., 2014). In addition, Borysiuk et al. (2018) demonstrated recently in *Arabidopsis thaliana* plants grown with ${\text{NH}}_{4}^{+}$ as sole N source much more sugar accumulation compared to those grown with ${\text{NO}}_{3}^{-}$, and they proposed the concomitant increase of MG as a possible cause of ${\text{NH}}_{4}^{+}$ toxicity (Borysiuk et al., 2018).

These results evidence that the deleterious effect of increased MG production also occurs in plants through enhanced glucose catabolism, and inevitably during conditions that generate excessive sugar flux. Indeed, this MG synthesis in plants also leads to AGEs production with deleterious effects on plant cell metabolism (Bechtold et al., 2009; Hasanuzzaman et al., 2017; Figure 1). Subsequently, plants, as well as other living organisms including human, have the so-called ‘glyoxalase system' as most important mechanism to protect DNA and proteins by converting MG into a non-toxic compound, D-lactate (Sousa Silva et al., 2013). The glyoxalase system encompasses glutathione-dependent glyoxalase I (GLXI), which converts MG to S-lactoylglutathione (Thornalley, 1990), and glyoxalase II (GLXII), which converts S-lactoylglutathione to D-lactate (Norton et al., 1989). Glyoxalase III (GLXIII) represents a glutathione-independent pathway that converts MG to D-lactate in a single step (Ghosh et al., 2016; Kumar et al., 2021), and has been previously reported in humans as well (Lee et al., 2012). Apart from the glyoxalase system, MG can be degraded by non-glyoxalase system in living systems including plants. It can be reduced by aldo-keto reductases or aldehyde dehydrogenases using NADPH to form lactaldehyde and finally pyruvate by lactate dehydrogenase (Takahashi et al., 2012; Saito et al., 2013).

## MG as a Reliable Ammonium Toxicity Indicator Which Can Be Cheap and Easily Detected

Since MG is a widespread molecule produced in stressful situations linked to an excess of mobile sugars in metabolism, it may also be considered as a reliable indicator of glycolysis overflow due to biotic and abiotic stresses in plants. ${\text{NH}}_{4}^{+}$-fed plants also show the *dicarbonyl stress* symptoms, making MG a good indicator for the degree of a ${\text{NH}}_{4}^{+}$ toxicity as well as a key agent to better understanding how it is generated, and especially mitigated to increase plant tolerance to ammonium-derived stress.

Among all the methods to quantify MG, the one described by Wild et al. (2012), and refined for plants by MacWilliams et al. (2020), has been found the most economical and safe way using a simple perchloric acid extraction process. The extracted supernatant is neutralized with potassium carbonate, and MG quantified through its reaction with N-acetyl-L-cysteine to form N-α-acetyl-S-(1-hydroxy-2-oxo-prop-1-yl)-cysteine, a product that is quantified spectrophotometrically at 288 nm (Wild et al., 2012; MacWilliams et al., 2020).

## Discussion

This opinion article uses the evidence of the excessive accumulation of MG by accelerated glycolysis observed under certain plant stresses to postulate why strict ${\text{NH}}_{4}^{+}$ could cause something similar to diabetes-like syndrome in plants by higher demand for sugars.

Despite the many studies that have explored why an excess of ${\text{NH}}_{4}^{+}$ in the absence of ${\text{NO}}_{3}^{-}$ can negatively affect plant growth, the ammonium syndrome and the role of ${\text{NO}}_{3}^{-}$ in counteracting it remains unclear and fully understood, so the present article creates a new paradigm shift targeting the generation of MG within the primary metabolism.

Finally, we consider that with in-depth research on the glycolysis alteration as by an excess of ${\text{NH}}_{4}^{+}$ nutrition, it is also possible to open new avenues for the improvement of plant stress tolerance through better N-management and genetic approaches. For instance, an interesting biotechnological strategy could be improving the effectiveness of the enzymatic and non-enzymatic cellular mechanisms responsible for maintaining low levels of MG. Also note that, in the future, photosynthesis and glycolysis in primary plant metabolism will be enhanced by the concomitant increase in atmospheric CO_2_ concentration and ${\text{NH}}_{4}^{+}$-based fertilization, which is more likely to promote MG accumulation in plants. Considering these aspects in plant research would avoid the potential negative impact of MG on the growth and production of crops, thus allowing a better adaptation to stress conditions intensified by anthropogenic factors.

## Author Contributions

MR-M wrote draft of the manuscript. IA edited and finalized the manuscript. Both authors contributed to the article and approved the submitted version.

## Funding

This work was supported by MINECO Programme: PID2019-107463RJ-I00/AEI/10.13039/501100011033 and the Regional Research and Development Programme of the Government of Navarre (call 2020_project HORTA0,0; PC106-107). MR-M received funding from fellowship through Public University of Navarra.

## Conflict of Interest

The authors declare that the research was conducted in the absence of any commercial or financial relationships that could be construed as a potential conflict of interest.

## Publisher's Note

All claims expressed in this article are solely those of the authors and do not necessarily represent those of their affiliated organizations, or those of the publisher, the editors and the reviewers. Any product that may be evaluated in this article, or claim that may be made by its manufacturer, is not guaranteed or endorsed by the publisher.
